# Emerging Role of Autophagy in the Development and Progression of Oral Squamous Cell Carcinoma

**DOI:** 10.3390/cancers13246152

**Published:** 2021-12-07

**Authors:** Yomna S. Abd El-Aziz, Lionel Y. W. Leck, Patric J. Jansson, Sumit Sahni

**Affiliations:** 1 Faculty of Medicine and Health, University of Sydney, Sydney, NSW 2006, Australia; ysal2128@uni.sydney.edu.au (Y.S.A.E.-A.); llec6616@uni.sydney.edu.au (L.Y.W.L.); patric.jansson@sydney.edu.au (P.J.J.); 2 Bill Walsh Translational Cancer Research Laboratory, Kolling Institute of Medical Research, St Leonards, NSW 2064, Australia; 3 Oral Pathology Department, Faculty of Dentistry, Tanta University, Tanta 31527, Egypt; 4 Cancer Drug Resistance and Stem Cell Program, University of Sydney, Sydney, NSW 2006, Australia

**Keywords:** autophagy, oral squamous cell carcinoma, autophagy inhibitors, anti-cancer therapy, cancer progression

## Abstract

**Simple Summary:**

Autophagy is a stress responsive process which involves degradation of damaged cellular organelles to replenish the cell with the biomolecules required for its growth and survival. It has been linked to different type of pathologies including cancer. There is an emerging role of autophagy in oral cancer progression, which indicates that autophagy could be a candidate for therapeutic targeting. This review discusses the role autophagy plays in cancer progression, including oral cancer, and autophagy targeting as a potential therapeutic approach.

**Abstract:**

Autophagy is a cellular catabolic process, which is characterized by degradation of damaged proteins and organelles needed to supply the cell with essential nutrients. At basal levels, autophagy is important to maintain cellular homeostasis and development. It is also a stress responsive process that allows the cells to survive when subjected to stressful conditions such as nutrient deprivation. Autophagy has been implicated in many pathologies including cancer. It is well established that autophagy plays a dual role in different cancer types. There is emerging role of autophagy in oral squamous cell carcinoma (OSCC) development and progression. This review will focus on the role played by autophagy in relation to different aspects of cancer progression and discuss recent studies exploring the role of autophagy in OSCC. It will further discuss potential therapeutic approaches to target autophagy in OSCC.

## 1. Introduction

Oral Cancer is the sixth most common type of cancer worldwide [1]. Oral squamous cell carcinoma (OSCC) accounts for about 90% of oral cancer cases [2]. Surgery alone, or combined with radio- or chemo-therapy, is the major treatment options for OSCC patients [3]. However, OSCC patients still have a poor prognosis exemplified by the high recurrence rate, metastasis and development of drug resistance [4]. This highlights the importance of identifying key molecules and pathways that drive OSCC progression. Autophagy had been recognized as a crucial pathway used by cancer cells to survive under stressful microenvironmental conditions, which aids cancer cells in avoiding death by chemotherapeutic agents, which invade the surrounding tissues and metastasize [5]. Thus, targeting autophagy could be an effective therapeutic strategy against cancer. Recent studies indicate that autophagy has an important role in OSCC progression. However, further studies are required to comprehensively elucidate the role of autophagy in OSCC progression and to determine the potential of targeting autophagy as a viable therapeutic approach.

## 2. Autophagy

Autophagy is a complex cellular process by which cells degrade old and defective cellular components to meet their metabolic needs [6]. Under normal physiological conditions, basal levels of autophagy are critical to maintain the cellular homeostasis, development and metabolic balance [7]. It is also considered as a cellular adaptive response against different stressful cellular stimuli such as hypoxia, nutrient and energy deprivation, indicating its cytoprotective role [8]. Autophagy allows the cells to survive under harsh conditions by recycling unfolded proteins or damaged organelles to supply the cells with essential building blocks such as amino acids [9]. Apart from its pro-survival role, autophagy is also suggested as a mechanism of cell death, a process known as autophagic cell death (ACD) [10]. In the past, autophagy was considered as a bulk degradation process, but studies in the last two decades have demonstrated it to be a tightly regulated and highly selective process [11]. Autophagy dysregulation has been implicated in many pathologies such as neurodegenerative diseases, infectious diseases and cancer [12].

Three types of autophagy have been identified: **(1) Macro-autophagy:** This type of autophagy involves formation of double membrane vesicles called autophagosomes to engulf old or damaged organelles or proteins. Autophagosome then fuses with lysosome resulting in cargo degradation by lysosomal hydrolases [13]. Macro-autophagy is the most common studied form of autophagy [13] and will be referred to as autophagy in this review; **(2) Micro-autophagy:** This involves invagination in lysosomal membrane to engulf the cargo for degradation [14]; and **(3) Chaperon mediated autophagy (CMA):** CMA involves selective degradation of soluble protein with KFERQ motif through recognition via heat shock protein 70 (HSP 70) and delivery to lysosomes for degradation via lysosome associated membrane protein 2A (LAMP2) [15].

### 2.1. Core Autophagic Machinery

**Figure 1:**
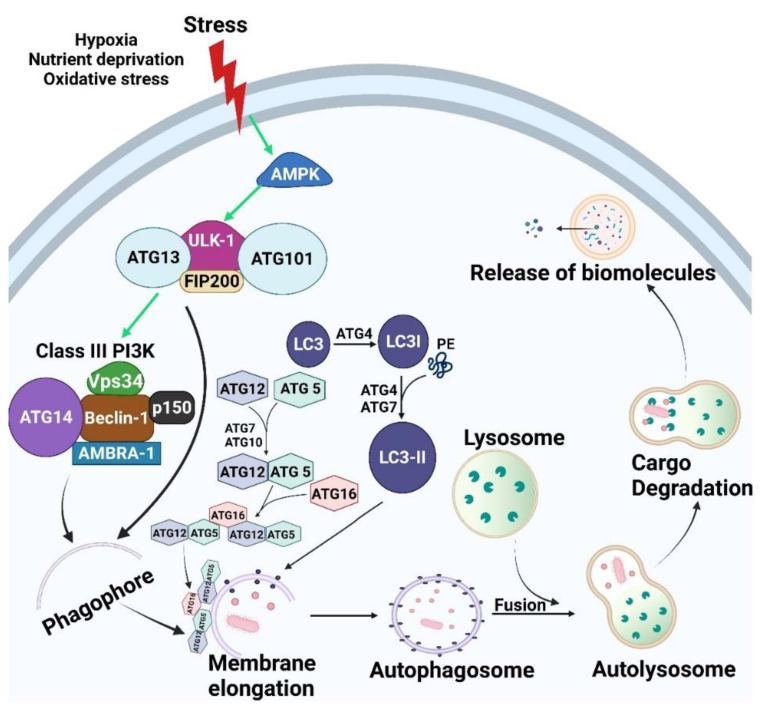
Autophagic pathway. As cells are subjected to micro environmental stress, AMPK activation occurs, which in turn leads to activation of ULK-1 complex (ULK-1, ATG13, ATG101 and FIP200). ULK-1 complex activation results in assembly of Class III PI3K (Beclin-1, Vps34, AMBRA, p150 and ATG14). Both ULK-1 complex and Class III PI3K translocate to the nucleation site and stimulate formation of the isolation membrane known as the phagophore. Elongation of the phagophore occurs via the effect of both LC3-II and ATG5-ATG12-ATG16 until a double membrane vesicle is formed, which is known as the autophagosome. Autophagosomes fuse with the lysosome which leads to cargo degradation via effect of lysosomal enzymes with release of biomolecules. Green arrows indicate activation. Created with BioRender.com (accessed on 15 October 2021).

Autophagy is regulated by a series of proteins encoded with a group of autophagy related genes (ATGs). It is a multistep process, each step being controlled by a group of ATGs [16] (Figure 1).

#### 2.1.1. Initiation/Induction and Nucleation

Accumulation of damaged organelles and proteins results in increased cellular stress, which induces autophagy initiation [17]. Autophagy initiation begins by activation of Unc-51 like autophagy activating kinase 1 (ULK-1), which results in assembly of a complex that contains ULK-1, ATG13, ATG101 and FIP200 [18]. ULK-1 complex activates class III phosphoinositide 3-kinase (PI3K) complex, which is comprised of Vps34, p150, Beclin-1, ATG14L, and Autophagy and Beclin1 Regulator 1 (AMBRA1) [19]. Both ULK-1 and Beclin-1 complexes localizes to the nucleation site and induce the emergence of a small membrane called phagophore, followed by the recruitment of downstream molecules involved in the next step [20].

#### 2.1.2. Elongation

The expansion of the phagophore is modulated via two ubiquitin-like conjugation systems, namely, ATG12-ATG5-ATG16 and microtubule-associated protein 1 light chain 3 (LC3) [17]. ATG12 is activated via ATG7/ATG10, followed by sequential conjugation with ATG5 and ATG16 [21]. This complex induces the elongation of the phagophore until a complete double membrane vesicle, named the autophagosome, is formed where ATG12-ATG5-ATG16 dissociate from the autophagosome’s membrane [21]. The second conjugation system involves LC3 processing. It starts with cleavage of precursor LC3 into LC3-I by ATG4. LC3-I is then conjugated with phosphatidylethanolamine (PE) forming LC3-II, which aids in membrane elongation [22]. This latter lipidation process is mediated by ATG4 and ATG7. In contrast to ATG12-ATG5-ATG16, LC3-II remains on the autophagosome membrane after its complete closure and thus is the most commonly used classical autophagosome marker [23].

#### 2.1.3. Maturation/Degradation

In this stage, the autophagosome fuses with endo-lysosomal compartment to form autolysosomes, which degrade the cargo via acidic lysosomal hydrolases and ends by release of biomolecules into the cytoplasm for reuse [24].

There is increasing evidence that autophagy is a highly selective process, as cargo recognition and targeting is performed through specific autophagy adaptor proteins that recognize the cargo and direct it toward the autophagosome for further lysosomal degradation [17]. p62 is an autophagic adaptor protein that recognizes mono or poly-ubiquinated proteins via its LC3 interacting region, which binds to LC3, and a C-terminal ubiquitin associated (UBA) domain that attaches to ubiquinated proteins [25]. Eventually, p62 is degraded together with its cargo within the lysosomes [25]. Hence, LC3-II and p62 are commonly used to measure the autophagic flux [23]. Bcl2-interacting protein 3-like (BNIP3L), next to BRCA1 (NBR1), calcium-binding and coiled-coil domain-containing protein 2 (CALCOCO2), and optineurin (OPTN) are other selective adaptor proteins that have been identified as delivering cargo to autophagosomes [16].

### 2.2. Mechanisms Regulating Autophagy

**Figure 2:**
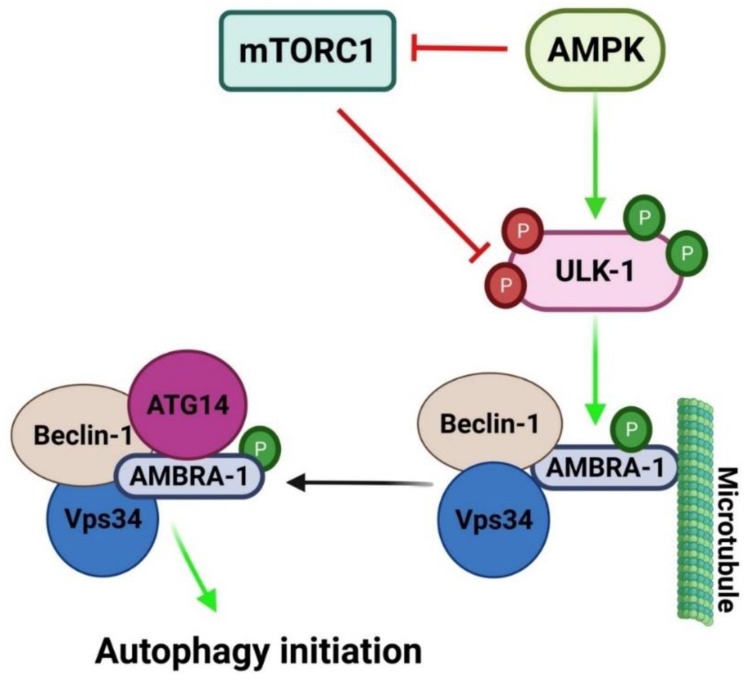
Regulatory mechanism for autophagic initiation. Under stressful micro environmental conditions, AMPK is activated, which in turn induces autophagy initiation either by inhibiting mTORC1 or direct phosphorylation of ULK-1 at Ser317 and Ser777. Activated ULK-1 phosphorylates AMBRA-1 that is attached to Beclin-1/Vps34 and results in its dissociation from the microtubule. This activates the assembly of ClassIII PI3K leading to autophagy initiation. mTORC-1 negatively regulates autophagy through inhibition of ULK-1 via phosphorylation at Ser757. Green arrow indicates activation, red arrow indicates inhibition and P indicates phosphorylation. Created with BioRender.com (accessed on 15 October 2021).

The autophagic pathway is controlled by various molecules such as mammalian target of rapamycin (mTOR), adenosine monophosphate-activated protein kinase (AMPK), ULK-1 complex and Class III PI3K complex (Figure 2) [17]. mTOR is involved in two complexes; mTORC1 and mTORC2, but only mTORC1 is it a negative regulator of autophagy [26]. Under nutrient rich conditions, mTORC1 phosphorylates ULK-1, at Sr757 phosphorylation site, rendering it inactive [8]. In contrast, under nutrient deprived stressful conditions, mTORC1 is inhibited, releasing ULK-1 from phosphorylation. This results in ULK-1 activation [16] with subsequent formation of a complex with ATG13, ATG101 and FIP200. The complex then transfers to the isolation membrane site and induces nucleation [17]. ULK-1 complex also induces assembly of Class III PI3K complex which is crucial for autophagy induction [18].

AMPK is a serine/threonine protein kinase which acts as a sensor of energy levels in the cell [27]. When low energy levels are encountered within the cell, as in case of glucose starvation, AMPK is activated which in turn induces autophagy initiation either by inhibiting mTORC1 or direct phosphorylation of ULK-1 at Ser317 and Ser777 which leads to initiation of autophagy [28].

## 3. Crosstalk between Autophagy and Apoptosis

Autophagy and apoptosis are pivotal processes that regulate the turnover of cellular components and cell death, respectively. There is an intricate interaction between these two processes [29,30,31,32]. Some regulatory proteins such as death associated protein kinase (DAPK), c-Jun N-terminal kinase (JNK) and BH3-only proteins (BAD, BID) are involved in the interplay between both autophagy and apoptosis [33]. Bcl2, one of anti-apoptotic proteins, has a BH3 domain groove which is occupied by the BH3 domain of Beclin-1. Due to the formation of Bcl2-Beclin-1 complex, Bcl2 exerts an inhibitory effect on both autophagy and apoptosis [34]. This interaction is interrupted by BH3-only proteins, which bind to Bcl2 and release Beclin-1 from Beclin-1-Bcl2 complex resulting in induction of autophagy [35]. These BH3-only proteins have also been shown to have a pro-apoptotic role [36]. Further, Bcl2-Beclin-1 complex can be disrupted through phosphorylation of either Beclin-1 or Bcl2 via DAPK and JNK, respectively, leading to liberation of Beclin-1 from the complex, permitting autophagy commencement [37,38].

## 4. Autophagy and Ferroptosis

Ferroptosis is a non-apoptotic, iron dependent form of cell death [39]. According to Dixon and Stockwell, ferroptosis is defined by three essential hallmarks; namely: (1) loss of lipid peroxide repair capacity by the phospholipid hydro-peroxidase GPX4; (2) availability of redox-active iron; and (3) oxidation of polyunsaturated fatty acid (PUFA)-containing phospholipids [39]. Emerging evidence has demonstrated a significant link between ferroptosis and autophagy. Activation of autophagy has been shown to be required for the induction of ferroptosis through the degradation of ferritin [40,41]. In glioblastoma (GBM), the autophagy inhibitor, quinacrine, enhanced GBM stem cells sensitivity to temozolomide via ferroptosis-mediated cell death [42]. Another study demonstrated autophagy as a positive regulator for ferroptosis through ferritinophagy, which is a form of cargo-specific autophagy [43]. Upon cysteine deprivation, autophagy is activated to degrade ferritin which is iron storage protein [43]. This process is known as ferritinophagy and is mediated by specific cargo nuclear receptor co activator 4 (NCOA4) [43]. This results in maintenance of cellular labile iron pool which in turn leads to reactive oxygen species (ROS) accumulation within the cell and consequently ferroptosis [43]. Indeed, genetic or pharmacological inhibition of autophagy and *NCOA4* knockdown abrogated cellular labile iron and ROS, as well as eventual ferroptosis cell death [40,43]. Further, the putative gene, *TP53*, which plays important role in the regulation of autophagy, also appears to mediate ferroptosis. Jiang et al. [44] demonstrated that p53 inhibited cysteine uptake and consequently sensitized cells to ferroptosis.

Recently, several studies have explored the role of ferroptosis in OSCC. For instance, Li et al. [45] identified several ferroptosis-related genes (FRGs) in OSCC specimens as promising prognostic biomarkers and these markers include *ATG5*, *MAP1LC3A* and *MAP3K5*, which also happen to be crucial genes in regulating autophagy. Another study has identified the ferroptosis-related gene- signature (FP score) and showed that high FP score correlated with longer survival rate and immune activation [46]. Furthermore, ferroptosis was suggested as a possible mechanism that kills OSCC cells when exposed to non-thermal plasma (NTP), a form of atmospheric-pressure plasma exposure [47]. After OSCC cells’ exposure to NTP, lipid peroxidation occurred and mitochondrial super-oxides were generated which are signs of ferroptosis [47]. These studies indicate potential key interactions between both autophagy and ferroptosis, which may provide a promising platform for optimizing combination therapy in cancer.

Several inducers for ferroptosis have been developed over the years. The RAS Selective Lethal small molecule 3/5 (RSL3/5) was one of the earliest molecules to be developed to target ferroptosis [48]. Combination of paclitaxel with RSL3 induced ferroptosis-related cell death in hypopharyngeal squamous cell carcinoma [49]. Several other promising FDA-approved ferroptotic-inducer compounds, such as erastin, altretamine, salazo-sulfa-pyridine and sorafenib have been developed [50,51,52]. While these compounds have been tested in several solid tumors, studies of these compounds being evaluated in OSCC still remains elusive. Future research is needed to elucidate the efficacy of these ferroptotic reagents in OSCC. Nevertheless, these studies highlight that targeting ferroptosis via autophagy and vice versa may provide promising alternative strategies in inhibiting OSCC progression.

## 5. Autophagy and Cancer

Autophagy plays a complex, dual and paradoxical role in cancer. At early carcinogenesis, autophagy exerts a tumor suppressor function as it limits the DNA damage and genomic instability through induction of senescence, thus limiting tumor growth. However, in advanced cancers, autophagy also contributes to tumor progression by supporting metabolic reprogramming of cancer cells to survive under different stressful conditions [53]. In addition, autophagy is known to be regulated by some tumor suppressors (e.g., p53) and oncogenes (e.g., RAS) [54].

### 5.1. Regulation of Autophagy by Oncogenes and Tumor Suppressors

**(i) p53:** The tumor suppressor, p53, is known as the guardian of the genome as its main function is to activate cell cycle arrest or cell senescence in response to DNA damage. It is considered to be a tumor suppressor as it eliminates cells with mutated DNA from the cell cycle and stimulates apoptotic genes. This function allows p53 to maintain cellular homeostasis and prevents processes that lead to tumor transformation [55]. However, more than 50% of human cancer encounters p53 loss of function [56] due to its direct mutation or mutation in kinases responsible for its activation [57]. Recently, p53 was found not only to act as a transcription factor that control cell cycle [55] and pro-apoptotic effects [58], but also to regulate autophagy.

p53 has a dual role on autophagy according to its cellular localization [59]. In the nucleus, p53 acts as pro-autophagic factor as it stimulates the autophagy via transactivation of many genes responsible for autophagy activation. Nuclear p53 induces the activation of AMPK, which is an upstream regulator of autophagy [60]. It also activates DAPK-1 that in turn phosphorylates Beclin-1 and liberates it from Bcl2/Beclin-1 complex, resulting in activation of autophagic initiation [38]. p53 also triggers *ULK-1* and *ULK-2* transcription leading to elevated levels of autophagy [61].

In contrast, cytoplasmic p53 tends to repress the autophagic machinery. One of the p53 targets is the TP53-induced glycolysis and apoptosis regulator (TIGAR), which inhibits intracellular levels of reactive oxygen species (ROS). This inhibition of ROS leads to suppression of the autophagic process [62]. Furthermore, Morselli et al. [63] showed that cytoplasmic p53 could inhibit autophagy through its interaction with FIP200, which is one of the major molecules in autophagy initiation. Taken together, the dual function of p53 is in line with the paradoxical role of autophagy in cancer and depends on subcellular localization of p53. Thus, future studies should take this into consideration to determine whether p53 has pro- or anti-autophagic role under certain settings.

**(ii) *RAS*:***RAS* is an oncogene that is activated in cancer cells. It is responsible for regulation of cell survival and growth [64]. Recently, several studies had linked *RAS* with autophagy, where autophagy could either enhance or suppress the oncogenic effect of *RAS* [35,65,66]. When *RAS* expression was acutely induced, which mimics the early stage of carcinogenesis, this resulted in induction of autophagic cell death via upregulation of Beclin-1 [35]. Another study showed that, in response to *RAS* activation, autophagy induces tumor cells to acquire a senescent state so as to limit the tumor cell proliferation [67].

In other experiments, stable exogenous over-expression of *RAS* resembles a phenotype corresponding to later stages of cancer progression [65,68,69]. In this context, autophagy is induced to allow metabolic reprogramming of cancer cells to deal with metabolic stresses, which promotes tumor cell survival [65]. As *RAS* hinders Acetyl CoA production from pyruvate, which is the main substrate of mitochondrial tri-carboxylic acid (TCA) cycle, autophagy provides substrates as amino acids to recharge the mitochondrial cycle [70]. Additionally, *RAS* induced autophagy has been shown to remove damaged mitochondria from the cytosol and thus preserves the cellular homeostasis [54]. Moreover, studies have demonstrated that inhibition of autophagy in *RAS* expressing cancer cells resulted in accumulation of damaged mitochondria with impairment of oxidative phosphorylation and reduced tumorgenicity of *RAS* mutant cells [65,66]. Taken together, *RAS* induced autophagy has a binary effect on tumorigenic process according to the context and tumor stage.

**(iii) *MYC*:***MYC* is one of the oncogenes that is activated and contributes to many aspects of human cancer [71]. High levels of MYC induce proliferation of tumor cells and stimulate the transcription of genes involved in mitochondria biogenesis and glycolysis, thus promoting metabolic reprograming of cancer cells [72]. Therefore, *MYC* knockdown enhanced growth arrest and apoptosis in tumor cells both in vivo and in vitro [73,74]. Notably, it was demonstrated that MYC inhibited the autophagic pathway in human MYC driven B-cell lymphoma through suppressing the transcription of genes responsible for autophagy [75]. Similar results were observed in HeLa human cell line [76]. In contrast, Toh et al. [77] demonstrated that *MYC* knockdown in HeLa cells had an inhibitory effect on autophagy via regulating JNK-1 activity and phosphorylation of Bcl2. In the same context, Dey et al. [78] found that MYC induced endoplasmic reticulum stress mediated autophagy in human lymphoma cells. These data indicate a dual effect of MYC on autophagy, which needs further investigation and may be unveiled as a new therapeutic approach for MYC driven cancers.

### 5.2. Autophagy as a Tumor Suppression Pathway

Autophagy has been connected to tumor suppression via the discovery of monoallelic loss of autophagy gene *BECN1* in several types of human cancers such as breast and ovarian cancers [79]. In addition, *BECN1* heterozygous deletion leads to increased malignancies in mice [80], with decreased tumor growth when *BECN1* expression is restored [81]. Moreover, mutation of other autophagy related genes such as *ATG5*, *ATG 9* and *ATG 12* have been observed in gastric and colorectal cancers [82]. These data suggest that autophagy repress tumor initiation. 

Autophagy is involved in tumor suppression through several pathways. The main function of autophagy is to maintain cellular hemostasis through degradation of damaged organelles and protein aggregates [7]. Thus, autophagy helps to protect the cells against DNA damage and genomic instability caused by elevated levels of ROS. This damage and instability result in increased risk of tumor initiation [83]. Another mechanism via which autophagy mediates its tumor suppressor function is through induction of senescence. Senescence is a prolonged growth arrest process in which the cell is metabolically active, but cannot undergo further division or re-enter the cell cycle [84]. Autophagy prevents the proliferation of transformed cells via oncogene induced senescence [84]. Depletion of autophagy genes leads to escape of abnormal cells from senescence [67]. This indicates that autophagy exerts a role in acquiring senescent phenotype upon oncogene activation, leading to the elimination of abnormal cells from the cell cycle.

Moreover, autophagy can inhibit tumor development by controlling the cellular levels of p62. The main function of p62 is to deliver ubiquinated proteins to autophagosomes for degradation and subsequently p62 is being degraded in this process [85]. p62 tends to be involved in pro-tumorigenic signaling and has been overexpressed in human cancers [85]. It was found that p62 depletion decreased the tumor size in *ATG7* deficient mice [86] and eradicated RAS-induced lung carcinoma [87]. p62 is also considered as a regulator of NRF2, which is a transcriptional factor responsible for upregulation of antioxidant genes under oxidative stress. Normally, NRF2 binds to KEAP1 for its polyubiquitination and proteasomal degradation [88]. Under autophagy deficient conditions, p62 accumulates in the cytoplasm and binds to KEAP1 at the NRF2 binding site, which leads to NRF2 activation that allows cancer cells to survive under oxidative stress [70].

Another potential mechanism by which autophagy may contribute to tumor suppression is through prevention of necrotic cell death and subsequent inflammation that may drive tumor growth. Under apoptotic and autophagy deficiencies in the tumor xenograft model, necrosis and subsequent inflammation were increased, which resulted in accelerated tumorigenesis by the effect of a pro-tumorigenic inflammatory microenvironment [89]. Therefore, the anti-inflammatory activity of autophagy helps to eliminate apoptotic cells, which could limit the inflammation that may contribute to tumor growth [90].

### 5.3. Autophagy as a Tumor Promoting Mechanism

Beside its tumor suppressive role, autophagy has been shown not only to promote malignant transformation into cancer cells, but also to support the survival of cancer cells. Deletion of autophagic genes such as FIP200 or ATG5 or ATG7 abolishes tumor growth in mouse models [66,91]. Similarly, shRNA induced knockdown of ATG5 hinders pancreatic adenocarcinoma in a mouse model [66]. Moreover, Yang et al. [66] demonstrated that pancreatic cancer cells had elevated basal autophagy levels, which were required for sustained tumor growth. This group also showed that autophagy inhibition using chloroquine or RNAi led to impairment of tumor growth both in vitro and in vivo [66]. These studies reflect the vital role of autophagy in tumor maintenance and progression.

Autophagy can serve as pro-tumorigenic factor via different potential mechanisms. It is well known that cancer cells are metabolically stressed due to their rapid tumor growth and poor blood supply, which results in lack of nutrients and oxygen availability. As a compensatory mechanism, cancer cells up-regulate autophagy to provide themselves with the required nutrients for survival [92]. Autophagy induction in hypoxic regions is regulated by HIF-1, which stimulates BNIP3 that in turn disrupt the association between Beclin-1 and Bcl2 which consequently leads to release of Beclin-1 and induction of autophagy [93].

Furthermore, autophagy contributes to cell dormancy, which is a state in which cells stop their division and motility to preserve energy [84]. Dormancy is also known to play a crucial role in tumor recurrence [84]. Under microenvironmental stress, autophagy can sustain cell survival by supplying the required nutrients during the dormant state. When the microenvironment improves, cancer cells can reenter the cell cycle and start proliferation again [94]. This phenomenon allows cancer cells to utilize autophagy to cope under metabolic stresses, which later can result in tumor recurrence.

Conversion of normal cell to a malignant cell phenotype is associated with the cascade of molecular and biological changes that result in cancer initiation and progression [95,96]. These series of events were defined as “Hallmarks of cancer” and were first reported by Hanahan et al. [95]. Recently, pro-survival autophagy had been linked to these hallmarks of cancers as a positive regulator.

#### 5.3.1. Self-Sufficiency of Growth Signals—Sustained Proliferation

One of the major hallmarks of cancer is the autonomy of growth signals to sustain their proliferative ability [96]. To achieve this, cancers utilize autophagy to provide themselves with the building blocks and ATP required for various functions by recycling damaged organelles and nutrients [92]. Elevated oncogenes expression also results in increased levels of basal autophagy, that helps in tumor longevity under various stress conditions [97]. Thus, tumor cells highly depend on autophagy for their survival and growth, which has been described as “autophagy addiction” [98]. As an example of this phenomenon, pancreatic ductal adenocarcinoma with *KRAS* mutation shows higher basal autophagy levels, which promotes tumor growth and contributes to gemcitabine resistance in pancreatic cell lines [99]. Autophagy inhibition in pancreatic cancer cells also led to tumor regression [97]. In a similar manner, *ATG7* deletion in a lung cancer model inhibited tumor growth [100]. Moreover, autophagy is important for non-small cell lung cancer (NSCLC) growth by decreasing ROS levels [101]. These data indicate that autophagy is essential for continued growth of cancer cells.

#### 5.3.2. Evasion of Apoptosis

Apoptosis is the programmed cell death cascade in multicellular organisms [102]. In case of cancer, tumor cells have deficient apoptotic machinery which contributes to sustained proliferation of cancer cells even after DNA damage and oncogene activation [96]. It has been well established that p53, which induces apoptosis when DNA damage occurs beyond repair, is lost in different types of cancer. The loss of p53 confers cancer cells with resistance against apoptosis [95].

Autophagy and apoptosis are closely related to one another and a crosstalk exists between these two cellular machineries as they have many common core mediators and upstream regulators [103]. As autophagy has been shown to have a dual role in cancer, it can resist or enhance the apoptosis according to the intensity of the stimulus and the threshold of each response [104]. For instance, Abedin et al. [105] demonstrated that Beclin-1 and ATG7 mediated autophagy suppress apoptosis after DNA damage and extended the survival of breast cancer cells. In line with the previous data, knockdown of *ATG7* resulted in enhanced apoptosis in melanoma cell lines [106]. Of note, mitochondrial autophagy was found to have a protective role against heat shock induced apoptosis via inhibiting the release of cytochrome C and caspase 3 [107]. In the same context, Das et al. [108] demonstrated that activation of a selective type of autophagy (i.e., mitophagy) in response to benzo(a)pyrene treatment, major component of cigarette smoke and vehicle exhaust, could rescue human immortalized skin epithelial keratinocytes (HaCaT cells) from benzo(a)pyrene–induced apoptosis. Inhibition of autophagy via chloroquine also promoted bevacizumab-induced apoptosis in colorectal cancer cells [109]. Similar results were found in pancreatic cancer cells as autophagy suppression by chloroquine induced doxorubicin mediated apoptosis [110].

On the other hand, autophagy may also be implicated in cell death, namely “autophagic cell death”. It is a form of cell death that is caspase independent and happens due to excessive autophagy [111]. Autophagic cell death can be induced by the effect of certain chemotherapy regimens as in case of glioblastoma where it was demonstrated that chemotherapy led to autophagic cell death and tumor regression [112]. Notably, it was demonstrated that Emodin, a Chinese herb, induced apoptosis in colon cancer cells through stimulation of autophagy [113]. The same observation was noted in hepatocellular carcinoma cells (HepG2 and Huh7) where autophagy inhibition via 3-methyladenin inhibited apoptosis was caused by isoquercitrin (ISO) exposure [114]. Collectively, these findings reflect a potential role of autophagy in regulation of apoptosis during tumor progression.

#### 5.3.3. Angiogenesis

Angiogenesis means the ability of tumor to form a new vasculature. Tumor cells cannot grow more than few millimeters due to lack of blood supply. That is why cancer cells begin to switch from a vascularized state to vascularized state, often called “angiogenic switch” [115]. Cancer cells stimulate themselves, or stimulate other cells to, secrete vascular endothelial growth factor (VEGF) to facilitate endothelial cells to form new blood vessels [116]. Autophagy has been implicated to play a role in angiogenesis. Du. et al. [117] demonstrated that ATG5 modulate angiogenesis in endothelial cells via AKT activation under hypoxia and starvation. Moreover, high mobility group box protein (HMGB) is a nuclear protein that has sequence homolog with Beclin-1, stimulates autophagy [118]. HMGB1 acts as an inflammatory cytokine that induce angiogenesis both in vivo and in vitro [119]. These findings indicate a role of autophagy in angiogenesis, but more research is needed to elucidate the regulatory mechanism exerted by autophagy in regard to angiogenesis.

#### 5.3.4. Invasion and Metastasis

One of the properties that cancers gain during their malignant transformation is the ability to invade surrounding tissues and settle down away from the primary site, which is known as “metastasis” [95]. It is a multistep process that involves: (1) Local invasion of the surrounding tissues; (2) Intravasation in the circulation; (3) Survival of cancer cells in the circulation; (4) Extravasation at secondary site; (5) Cancer cells survival at the secondary site; and (6) Tumor development at the secondary site [95].

It has been shown that autophagy has an integral role in metastasis, as autophagy mediates an important role in epithelial to mesenchymal transition (EMT) [120]. Autophagy is also involved in persistence of dormant cancer cells in the circulation and at the secondary site [121]. Various studies have linked autophagy to invasion and metastasis as a positive regulator. Silencing of *ATG12* and *SQSTM1* decreased invasion and metastasis of glioma cells in 3D cell culture model [122] and glioblastoma stem cells [123] in vitro, respectively. In addition, starvation induced autophagy increases invasion and metastasis of hepatocellular carcinoma (HCC) via TGF-β/SMAD3 signaling [124]. A similar connection was observed in colon cancer where SMAD3 suppression via microRNA resulted in interrupted autophagy and reduction in invasiveness capability [125]. Moreover, autophagy inhibition via chloroquine or siRNA against *ATG3* or *ATG7* in HCC resulted in repression of EMT [124]. Taken together, autophagy is involved in every step of metastasis, which could be utilized as a therapeutic approach.

#### 5.3.5. Anoikis Resistance

Anoikis is programmed cell death that occurs due to loss of detachment with the extracellular matrix (ECM). Normal cells have integrins on their surface, which help to maintain their anchorage to the ECM and preserve tissue homeostasis [126]. In contrast, cancer cells are characterized by loss of integrins, which lead to their detachment, EMT transition and migratory ability by way of anoikis resistance [127]. Autophagy has been involved in anoikis resistance as cancer cells tends to upregulate autophagy to promote their survival under ECM detachment stress [127]. Avivar-Valderase et al. [128] demonstrated that autophagy is responsible for the survival of ECM detached mammary epithelial cells via activation of AMPK. Autophagy inhibition is also shown to reduce pulmonary metastasis in mouse model of hepatocellular carcinoma via impairing anoikis resistance [129]. Moreover, BNIP3 induced autophagy contributes to anoikis resistance in hepatocellular carcinoma [130]. Similarly, induction of autophagy by rapamycin enhances spheroid formation and survival in peritoneal carcinomas and sarcomas by boosting anoikis resistance and apoptosis inhibition [131]. In summary, autophagy is essential for survival of detached cancer cells, which represents the first step in invasion and metastatic process.

#### 5.3.6. Avoidance of Immune Destruction

Evasion of immune system is one of the major hallmarks in cancer [96]. Cancer cells tend to avoid immune destruction to allow their survival within the circulation until their extravasation and settlement at a secondary site [96]. Over the last few years, autophagy has been identified as one of the mechanisms in tumor immune surveillance. It was suggested that inhibition of tumor immune surveillance is induced by pro-survival autophagy in different types of cancers [132]. For example, lysis of MCF7 breast cancer cells by cytotoxic T-lymphocytes (CTL) was suppressed when they were induced to undergo EMT with concurrent induction of autophagy [132]. Autophagy inhibition in MCF7 cancer cells, through silencing of *BECN1*, restored the CTL lysis capability in the same model [132]. Impairment of CTL lysis in melanoma cells was also attributed to autophagy through degradation of Connexin 43 [133]. In the same manner, *FIP200* null cancer cells showed enhanced entrance of CTL within the tumor via elevated levels of CXCL10, which resulted in decreased primary tumor growth [91]. Notably, pancreatic cancer cells have reduced surface expression of major histocompatibility complex-1 (MHC-1) as it is lysosomally degraded via an autophagy dependent mechanism [134]. Furthermore, inhibition of autophagy boosted the destruction of renal cell carcinoma by natural killer cells [135]. Overall, autophagy seems to modulate tumor immune surveillance to allow the survival of disseminated tumor cells.

#### 5.3.7. Metabolic Reprogramming

Metabolic reprogramming is considered to be one of the hallmarks of cancer and is known as the “Warburg effect” [96]. Once cells have committed to malignant transformation, they seem to depend mostly on aerobic glycolysis for energy production and reduce the oxidative phosphorylation via mitochondrial electron transport chain [136]. This metabolic shift is induced by oncogenes such as *RAS* [137] and *MYC* [138]. As mentioned before, *RAS* transformation impairs production of acetyl-CoA from decarboxylation of pyruvate and induces the uptake of both glucose and glutamine [70]. *RAS* induced autophagy plays a crucial role in this shift by providing essential building blocks that fuels the reprogramming [69]. Although there is a dependence of cancer cells on aerobic glycolysis for energy production, they still rely on functional mitochondria for their growth. Autophagy provides amino acids, from degradation of proteins, which are required to replenish the TCA cycle [70]. Consistent with these data, some studies have shown that autophagy inhibition leads to impairment in glycolysis [69,91]. It has been reported that autophagy promoted glycolysis which is cardinal for RAS mediated oncogenic transformation [69]. Similarly, sustained glycolysis was observed in chronic myeloid leukemia with ATG7 mediated autophagy [139]. Taken together, these studies indicate the pivotal role of autophagy in metabolic reprogramming of cancer cells that needs to be further investigated to fully understand the underlying mechanisms and how it can be used as a therapeutic approach.

Overall, autophagy plays a major role in acquiring different properties of cancer cells which enable tumor growth and progression. Thus, autophagy is a good candidate for therapeutic targeting.

## 6. Autophagy in Oral Cancer

The role of the autophagic pathway has been studied in many types of cancer e.g., breast, pancreatic, prostate and ovarian cancer [80]. Recently, there has been emerging evidence of the critical role played by autophagy in the progression of oral cancer. This might open up new avenues for treatment which could potentially culminate in better outcomes for oral cancer patients.

### 6.1. Oral Squamous Cell Carcinoma (OSCC)

Oral cancer is considered to be the sixth most prevalent malignancy worldwide [1] with about 377,713 new cases diagnosed and 177,757 deaths annually [140]. Oral squamous cell carcinoma (OSCC) represents about 90% of all oral cancer cases [2]. The tongue is the most common site for OSCC followed by the lips [141]. Patients with oral cancer suffer from high morbidity and mortality rates and poor 5-year survival rates even after surgical excision. This is due to late detection, early local lymph node metastasis at time of diagnosis and local recurrence [4].

Surgical excision with safety margins is the most prevalent method for treatment of OSCC, but it can be combined with RT or CT before or after the surgery in the case of operable tumors, whilst RT or CT is used alone when the tumor is inoperable [3]. Cisplatin is the gold standard chemotherapeutic agent used for treatment of OSCC [142]. Administration of chemotherapeutic agents, e.g., Cisplatin, results in shrinkage of tumor size that facilitates RT feasibility and decreases the disfigurement associated with surgery [143]. However, such therapy has not increased overall survival (OS) in the past two decades [144]. This is usually due to development of chemo-resistance that reduces the efficacy of CT toward cancer cells [145]. Initial response towards the platinum based chemotherapies was satisfactory in OSCC patients, but 70% of the patients showed cancer relapse due to drug resistance [146]. Thus, OSCC still has a poor prognosis due to abrupt deterioration of oral health from surgery or RT and development of drug resistance to the anti-cancer agents [147]. Hence a better understanding of OSCC biology is required so that effective therapeutic targeting can be achieved to improve the survival rates and quality of life for patients.

### 6.2. Stressful OSCC Microenvironment

Previous research in OSCC had majorly focused on the cancer cells, their genetic alteration and how this affects their behavior and patient’s prognosis. However, there is increasing evidence about the inter-relation between cancer cells and surrounding tumor microenvironment (TME) components. TME contains different type of cells (e.g., cancer associated fibroblasts (CAFs), immune cells and endothelial cells) and extracellular matrix (ECM) that consists of collagen fibers, growth factors, cytokines and chemokines [148]. Thus, TME is considered a part of the cancerous tissue, which contributes to the behavior and prognosis of tumors [149].

#### 6.2.1. Cancer Associated Fibroblast (CAFs)

CAFs are non-immune, infiltrative cells that harbor in the tumor microenvironment [150]. The exact origin of CAFs is still debated. It is thought that they arise from normal resident fibroblast, the cancer cells themselves or bone marrow derived mesenchymal stem cells [151]. The trans-differentiation of normal fibroblast into CAFs seems to be regulated by molecules released from OSCC cells. An in vitro study revealed that OSCC cells produce tumor growth factor-β (TGF-β) that induces trans-differentiation of normal fibroblast into CAFs, which in turn secretes growth factors that result in OSCC proliferation and invasion [152]. Further, it was demonstrated that increased expression of IL-1β in OSCC results in activation of NFĸB in normal fibroblast that subsequently releases CXCL1 from the fibroblast [153]. CXCL1 creates an autocrine mechanism that causes transformation of normal fibroblast into CAFs [154]. CAFs are characterized by expression of α-smooth muscle actin (α-SMA) which is not found in normal fibroblast [155].

Clinico-pathological studies have shown that high stromal CAF existence is associated with poor prognosis in oral cancer [155,156]. CAFs are involved in multiple tumor activities such as sustained proliferation of tumor cells, angiogenesis, invasion and metastasis and actin cytoskeleton remodeling, which favors migratory properties through reciprocal crosstalk between cancer cells and CAFs [151]. Wong et al. [157] reported that increased expression of platelet derived growth factor receptor β (PDGFRβ) in CAFs led to activation of the JAK2/STAT3 pathway and release of epidermal growth factor (EGF) from CAFs to promote EMT. In the same context, it was demonstrated that OSCC cells release IL-1β, which stimulates the release of CCL7 from CAFs. The latter binds to CCR1-3, on the surface of OSCC, increasing cell migration in vitro [158]. These data indicate the role played by CAFs in the acquisition of migratory properties of OSCC cells.

Beside CAFs’ role in metastasis, they also exert an immunosuppressive role. It was shown that CAFs liberate anti-inflammatory mediators such as TGFβ and IL-10 that hinder T cell proliferation and increase infiltration of CD163 tumor associated macrophages (TAMs) [159]. CD163 M2 macrophages are usually expressed in the resolution stage of inflammation, indicating that their infiltration in TME activate an immunosuppressive environment [160].

Autophagy is known to play an important role in the mechanisms via which CAFs are known to promote tumor progression in a variety of cancers, such as pancreatic cancer [161,162]. Notably, Tan et al. [163] showed autophagy induction via TGF-β1 in normal oral fibroblast results in fibroblast activation and senescence, which are known to be associated with more aggressive tumors [164] and enhanced OSCC migration [163]. However, the exact role of autophagy in CAF mediated OSCC progression is currently elusive and is an important area for future research.

#### 6.2.2. Tumor Associated Macrophages (TAMs)

The main role of immune cells in TME is to help cancer cells evade immune destruction by secreting cytokines that block CTLs, which results in immune tolerance [165]. TAMs are one of the main immune cells in TME. CD163 M2 phenotype is the predominant type of TAM within TME [166]. TAMs are involved in many aspects of OSCC behavior. They can promote angiogenesis via secretion of angiogenic factors such as vascular endothelial growth factor (VEGF) [167]. They also induce proliferation of OSCC cells through epidermal growth factor (EGF) secretion [168]. Moreover, TAMs are involved in the invasion and metastasis process as NF-ĸB expressed by TAMs leads to upregulation of matrix metalloproteinases (MMPs), which remodel ECM in a way that permit an invasive and metastatic behavior [165]. It has been demonstrated that expression of CD163 TAMs is associated with poor prognosis and lymph node metastasis [169].

Interestingly, autophagy has been shown to play an important role in the polarization of macrophages to M2 phenotype in colon and liver cancer cells [170,171,172]. This polarization provides TME conditions conducive to tumor growth and progression, highlighting an essential role played by autophagy in TME.

#### 6.2.3. Hypoxia and Angiogenesis

As OSCC grows and reaches a size of a few millimeters, some tumor cells are not directly exposed to blood vessels in order to obtain nutrition, resulting in hypoxic microenvironment [173]. Hypoxia is the main triggering event that stimulates angiogenesis through upregulation of HIF-1α, which in turn induces expression of angiogenic factors such as VEGF [174]. It was found that HIF-1α is responsible for expression of multiple downstream targets that regulate different OSCC activities. Beside its role in angiogenesis, HIF-1α is also involved in proteolytic modification of ECM via stimulating the expression of proteolytic enzymes such as MMP-2 and cathepsin D, which are crucial for invasion and metastasis [175,176]. Moreover, HIF-1α facilitates EMT by inducing SNAIL and TWIST pathways that repress E-cadherin, which result in tumor metastasis [176,177].

It is well known that hypoxia is one of the conditions that trigger autophagy [178]. Under hypoxic conditions, activation of HIF-1α led to activation of BNIP3 that binds to Bcl2 and allow its release from the Bcl2-Beclin1 complex resulting in liberation of Beclin-1 and autophagy induction [179]. *HIF-1α* knockout have been shown to result in decreased levels of autophagic marker such as LC3 and Beclin-1 [180]. Another study revealed via gene expression data that hypoxia induced autophagy in the tumor initiating cells (TICs) of patient derived colorectal cancer culture [181]. In the same context, Yang et al. [182] demonstrated that activated autophagy in bladder cancer cells under hypoxic conditions significantly increase gemcitabine cytotoxicity when combined with an autophagy inhibitor (3-methyladenine). These data indicate that autophagy can be utilized by tumor cells in order to cope with a hypoxic TME.

#### 6.2.4. ECM

Collagen fibers are the main component of the ECM part of TME [183]. Modification of ECM occurs during different stages of the tumor. It was found that ECM of OSCC is characterized by increased stiffness due to collagen deposition by either cancer cells or stromal cells [184]. Increased ECM deposition was associated with poor prognosis and metastasis [185]. At the invasive front of OSCC, increased turnover of ECM was prominent, indicating increased EMT and high levels of crosstalk between cancer cells and stromal cells [186]. Increased expression of tenascin-1 and type I collagen were also observed in OSCC specimens with lymph node metastasis [187]. Importantly, recent studies have shown that autophagy is regulated by stiff microenvironmental conditions in breast cancer cells [188]. The effect of a stiff OSCC microenvironment on autophagy is still elusive and requires further investigations.

In summary, OSCC cells are subjected to different types of microenvironmental stressor which affect OSCC behavior. As autophagy is a stress response pathway, it appears to play a crucial role in the crosstalk between tumor and different TME components in OSCC. 

### 6.3. Role of Autophagy in OSCC Progression

OSCC, like any other type of cancer, develops in a multistep process that results from interaction between genetic and environmental factors, which eventually leads to oncogenesis and cancer progression [189]. Autophagy is one of the mechanisms that may contribute towards initiation and growth of the cancer cells, as seen in other types of cancers. Hence, autophagy could be an attractive target for the development of effective therapeutic strategy against OSCC [53].

Several studies have shed light on the possible role of autophagy in OSCC, which may contribute to tumor progression and prognosis. De et al. [190] reported a significant increase in levels of autophagy in OSCC specimens, through quantification of LC3 immuno-positive cells by immunohistochemistry (IHC), in comparison to normal mucosa and premalignant lesions e.g., leukoplakia. Similarly, LC3 expression by IHC was correlated with unfavorable prognosis in OSCC specimens [191]. The same group demonstrated that ATG9A overexpression was associated with poor overall survival and earlier relapse [192]. Another study showed that ATG16L1 upregulation is related to shorter overall survival and a more aggressive phenotype [193]. In the same manner, Liu et al. [194] observed that increased LC3 expression is linked to aggressive clinicopathological features. However, it should be noted that LC3-II, not LC3, is a marker for autophagosome. Furthermore, the levels of autophagosomes in the cells are balanced between autophagic initiation and lysosome mediated degradation. Hence, future studies examining simultaneous expression of multiple molecules in autophagic machinery are necessary to further delineate the role of autophagy in OSCC progression.

Interestingly, overall survival rate of OSCC patients with high p62 expression post chemotherapy was lower than those with low p62 expression [195]. Although p62 is involved in shuttling cargo, it degrades with the cargo when autophagosomes fuse with lysosomes. Therefore, evaluation of p62 levels needs to be assessed at both mRNA and protein levels. Liu et al. [194] demonstrated that high *SQST*M1 (i.e., gene encoding p62) mRNA expression correlated with high p62 protein expression in the cytoplasm, which was associated with aggressive clinicopathologic features and unfavorable prognosis.

Additionally, autophagy is suggested to be involved in protection of OSCC cells from apoptosis and allow their survival under different microenvironmental stresses. Park et al. [196] showed that fisetin, a natural flavonoid that has anti-oxidant activity, induced apoptosis of OSCC cells through inhibition of autophagy. Furthermore, Lin et al. [197] showed that ursolic acid induced autophagy in an OSCC cell line (Ca922) and autophagy inhibition by chloroquine increased ursolic acid induced apoptosis. This latter study provides further evidence of the protective role played by autophagy in OSCC.

It was observed that circCDR1 enhanced the viability of OSCC cells under hypoxic conditions through autophagy induction [198]. Likewise, clusterin induced autophagy in oral cancer cell lines that resulted in cell survival and protection from serum starvation induced apoptosis [199].

Autophagy also seems to play a role in chemotherapy resistance and cancer stemness. Naik et al. [200] showed that cisplatin resistant cell lines had higher levels of stemness markers and more autophagic flux than parental OSCC cells. When autophagy is inhibited, reduction in stemness was observed [200].

On the other hand, low expression of *BECN1* in OSCC specimens has been reported by multiple studies [201,202,203] and is associated with poor prognosis [201] and enhanced proliferation, invasion and metastasis [203]. Moreover, *BECN1* silencing was found to induce proliferation, migration and invasion of OSCC, while repressing apoptosis and restoring chemo-sensitivity to cisplatin [204]. These latter findings are in direct alignment with the tumor suppressor role of *BECN1* [205]. Importantly, Kong et al. [206] demonstrated that autophagy inhibited the invasive potential of OSCC via regulation of the NF-ĸB pathway. Furthermore, a study unveiled that curcumin induce apoptosis in OSCC cells via induction of autophagy, indicating that autophagy acts as pro-death signal [207]. Another study also exhibited tanshinon IIA induced autophagic cell death in OSCC cell line (SCC9) via induction of Beclin-1/Atg7/Atg12-Atg5 pathway [208]. Similarly, curcuminoids decreased cell viability in oral cancer cells by induction of autophagic cell death [209].

Taken together, there is increasing evidence about the crucial role of autophagy in OSCC progression, suggesting that autophagy has great potential as a future therapeutic target.

## 7. Targeting Autophagy as a Therapeutic Approach

**Figure 3:**
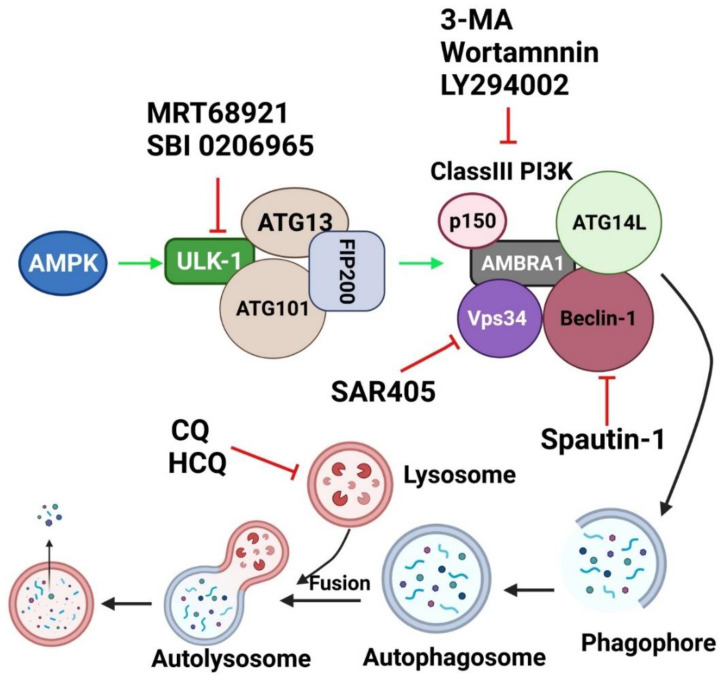
Autophagy Inhibitors and their site of action in autophagic machinery. MRT68921 and SBI-0206965 are ULK-1 inhibitors. 3-MA, Wortmannin and LY294002 are Class III PI3K inhibitors. Spautin-1 is a Beclin-1 inhibitor and SAR405 is a Vps34 inhibitor. Chloroquine (CQ) and hydroxyl chloroquine (HCQ) are late autophagy inhibitors which target lysosomal pH rendering the fusion between autophagosome and lysosome. Green arrow indicates activation and red arrow indicates inhibition. Created with BioRender.com (accessed on 15 October 2021).

Autophagy seems to play a pivotal role affecting all major hallmarks of cancer progression. Therefore, autophagic machinery can be targeted using different compounds that can inhibit the autophagic process at different stages of this complex pathway (Figure 3).

### 7.1. Chloroquine and Its Derivatives

Lysosomes are essential organelles in the autophagic pathway. Lysosomes fuse with the autophagosome leading to degradation of autophagosome content via their hydrolytic enzymes [210]. The function of lysosomal hydrolytic enzymes is dependent on the acidic environment within lysosomes (pH range 4.5 to 5) [211]. Over recent decades, many compounds have been developed to target the lysosome, which consequently also targets autophagy. Chloroquine (CQ) has been found to inhibit autophagy [212]. It is an antimalarial drug [213], which is also used in some autoimmune diseases such as rheumatoid arthritis [214] and lupus erythrematosus [215]. CQ becomes entrapped and protonated within the lysosome, which results in increased lysosomal pH that consequently renders lysosomal hydrolytic enzymes inactive [213]. Hence, it blocks autophagy at its late stage [216]. Although, CQ is well tolerated in humans, it causes retinal toxicity [217]. A CQ analogue, hydroxychloroquine (HCQ), has been developed by adding a hydroxyl group to CQ. This addition of hydroxyl group limits CQ’s capability to cross the blood–retinal barrier, thereby decreasing the retinal toxicity associated with CQ [218].

Over the last few years, CQ and HCQ have been used in preclinical studies as a combination therapy together with the conventional therapies. CQ/HCQ increase sensitivity of glioma cancer cells to radiation therapy and temozolomide treatment [219,220,221]. Likewise, the efficacy of mTOR inhibitor (e.g., everolimus), tyrosine kinase inhibitor (e.g., MK2206) and gefitinib is enhanced when co-administered with CQ/HCQ [222,223,224]. CQ is also shown to re-sensitize endometrial cancer cells to cisplatin [225] and overcame resistance to different chemotherapeutic agents such as cyclophosphamide [226], vorinostat [227], and saracatinib [226]. Further, HCQ enhances T cells/NK cells cytotoxicity in a murine model [228].

However, it had been found that CQ/HCQ may cause kidney impairment, and neurodegenerative and cardiovascular disease when administrated in high doses [229,230]. Despite this, several clinical trials have been performed to determine the efficacy of CQ/HCQ in combination with various standard chemotherapeutic agents against different types of cancer [231,232,233,234,235,236,237]. However, none of these trials have reached phase III, suggesting the necessity for the development of more selective compounds to inhibit autophagy. 

As CQ/HCQ may not be able to potently inhibit autophagy at a tolerable clinical dose, new derivatives have been developed [238]. Lys05 is a new derivative of CQ/HCQ that accumulates more readily in the lysosome, and has been demonstrated to increase the lysosomal pH and blocks autophagy at late stage [239]. Lys05 exhibited a 10-fold potency to block autophagy compared to HCQ [239]. Lys05 also displayed anti-tumor activity in mice without causing any non-specific toxic side effects [238]. Since, Lys05 has shown promising results in preclinical studies, a clinical trial of this agent would be of a great interest.

### 7.2. Specific Autophagy Inhibitors Directed toward Major Molecules in Autophagic Machinery

Recently, many autophagic inhibitors have been developed against major molecules or pathways involved in the early stage of autophagy. Most of these compounds are still at the preclinical stage and need further investigations to validate their potential under clinical settings [240].

#### 7.2.1. PI3K Inhibitors

Due to its pivotal implication in autophagy, PI3K has become an interesting target for autophagy inhibition. Phosphoinositide 3-kinases (PI3K) are divided into three groups, Class I, Class II and Class III. Class III PI3K corresponds to Vps34 and is responsible for autophagy induction, whilst, Class I PI3K triggers mTOR, which in turn inhibits autophagy [241]. Agents that have been developed to inhibit PI3K include 3-methyladenine (3-MA), Wortmannin and LY294002 [238]. 3-MA was first discovered from isolated hepatocytes of starved rats [242]. It is directed toward Vps34 of Class III PI3K, which results in autophagy inhibition [243]. However, prolonged administration of 3-MA can also inhibit Class I PI3K even in nutrient rich conditions. Hence, 3-MA appears to have a dual role in autophagy [244]. Despite this, high concentration of 3-MA is needed to inhibit autophagy in vitro, which results in inhibition of other kinases such as MAPK, mTOR and DNA-defendant protein kinase (DNA-PK) [245]. Moreover, beside lack of specificity and off-target activities, 3-MA also has poor solubility [238]. As a result, the clinical application of 3-MA is limited.

Wortmannin, derived from fungal metabolites [246], is another PI3K inhibitor that has anti-inflammatory activity [247]. Wortmannin is a highly potent inhibitor that inhibits Class III PI3K permanently, with transient inhibition of Class I PI3K, and has an inhibitory effect on other kinases [244]. Similarly, the PI3K inhibitor, LY294002, has a low potency against Class I and III PI3K, but affects more mTOR and DNA-PK pathways [248]. Hence, these three inhibitors have many limitations, including off-target activities, limited potency and solubility, which contribute to their limited clinical application.

Thus, various inhibitors have been designed to actively target Vps34 either directly (e.g., SAR405) or indirectly (e.g., Spautin-1). Vps34 is a member of the phosphoinositide 3 kinase (PI3K) protein family and a major molecule in autophagy induction. Vps34 converts phosphatidylinositol into phosphatidylinositol-3-phosphate (PI3P) via its lipid kinase activity, which is essential for autophagosome biogenesis [241].

Spautin-1 is a quinazolin derivative of 4-[[3,4-(methylene-dioxy)benzyl]amino]-6-chloroquinazoline (MBCQ) [249], which inhibits two ubiquitin specific peptidases, USP10 and USP13 [250]. These enzymes deubiquitinate Beclin-1 [250]. Under nutrient deficient conditions, treatment with Spautin-1 led to Beclin-1 ubiquitination and its proteasomal degradation, which in turn resulted in destabilization and degradation of Vps34 and consequently resulted in autophagy inhibition [250]. Thus, Spautin-1 indirectly inhibits Vps34 activity through Beclin-1 degradation. In preclinical studies, Spautin-1 shows synergism with Imatinib in chronic myeloid leukemia. Furthermore, Spautin-1 promotes cancer cell death under nutrient deficient conditions [251].

SAR405 is a potent and highly selective Vps34 inhibitor that directly affect the catalytic activity of Vps34 [252]. SAR405 suppressed autophagy under nutrient starvation [252] when combined with an mTOR inhibitor (e.g., AZD8055) [253]. Interestingly, synergism is also observed between SAR405 and other chemotherapeutic agents in different cancer models [254,255,256].

PIK-III is a bisamino-pyrimidine that is designed to selectively inhibit Vps34 by direct binding to the hydrophobic pocket at the ATP binding site of Vps34 [257]. PIK-III showed 100-fold better potency in Vps34 inhibition than Class I PI3K [257]. Despite the emergence of these Vps34 inhibitors, more preclinical studies are required to evaluate the potential of these inhibitors as clinically useful drugs.

#### 7.2.2. ULK-1/2 Inhibitors

ULK-1 is a key autophagy regulator that upon phosphorylation results in activation of multiple autophagy initiating proteins including Beclin-1 [258]. ULK-1 is subjected to negative and positive phosphorylation by mTOR and AMPK, respectively [259]. Various inhibitors, such as MRT68921 and SBI0206965, have been designed to target ULK-1 and lead to suppression of autophagy. MRT68921 is a potent highly specific ULK-1/2 inhibitor that acts via competitive ATP binding [260]. It exhibited off-target effect on kinases such as AMPK, but studies have demonstrated that MRT68921 reduced the autophagic flux via inhibition of ULK-1 specifically [260].

SBI-0206965 is a small molecule inhibitor of ULK-1/2 that acts through blocking the Vps34 phosphorylation by ULK-1 [261]. This agent suppressed autophagy induced by mTOR inhibitor AZD8055, resulting in apoptotic cell death [261]. SBI-0206965 also activates apoptotic pathway through destabilization of pro-survival proteins Bcl2 and BCLXL [262]. Recently, SBI-0206965 has also been identified as AMPK inhibitor [263,264]. These findings indicate that ULK-1 is a promising therapeutic target to inhibit autophagy.

Collectively, the new era in autophagy modulation, by using specific inhibitors directed toward major molecules of autophagic machinery, needs more preclinical investigations to validate these inhibitors as clinical useful agents.

## 8. Autophagy Inhibitors in OSCC

Some studies had been performed to evaluate the effect of autophagy inhibition in OSCC progression. These autophagy inhibitors target the major molecules in the autophagic machinery either in the early or late stage of the autophagic process. 

In regard to late stage autophagy blockage, autophagy inhibition by chloroquine showed a synergism with interferon-gamma with induction of apoptosis in OSCC cell lines and xenograft [265]. Ahn et al. [266] also observed enhanced cytotoxicity of Apicidin, a histone deacytlase inhibitor, in OSCC cells when combined with late stage autophagy inhibitor chloroquine. Similarly, chloroquine suppressed OSCC cell proliferation and colony formation ability, arrested the cell cycle in vitro and inhibited tumor growth in vivo [267]. In the same context, Magnano et al. [268] observed significant increase in the rate of apoptosis upon combination treatment of cisplatin and chloroquine compared to cisplatin alone in vitro, indicating that chloroquine facilitates re-sensitization of OSCC cells to cisplatin induced apoptosis. Additionally, blocking autophagy via bafilomycin-A1 enhanced the effect of baicalein, which is a phytochemical with inhibitory effect on cancer cells, inducing apoptosis. This latter study suggests a protective role of autophagy in OSCC [269].

In regards to early stage autophagy inhibitors, 3-MA was found to enhance apoptosis induced by nutrient depletion [270] and IL-24 [271]. Moreover, LY294002 loaded hyperbranched poly-acyl-hydrazone conjugated doxorubicin (LY-HPAH-DOX) micelles potentiated the inhibitory effect on proliferation of OSCC cells compared to HPAH-DOX or DOX alone [272]. Interestingly, co-administration of an early autophagy inhibitor SAR405 with cisplatin resulted in significant lower rates of apoptosis compared to cisplatin alone in OSCC cell lines, whilst combined treatment of cisplatin with late autophagy inhibitor CQ showed enhanced cisplatin induced cell death. This discrepancy may be due to the specificity of each inhibitor and may suggest that targeting autophagy at different stages may result in different cellular response [268]. Notably, autophagy inhibition via SAR405 induced normal oral fibroblast activation into myofibroblast [163]. 

Altogether, more pre-clinical research is still needed to elucidate if inhibiting autophagy is a viable therapeutic approach for OSCC treatment.

## 9. Conclusions

In summary, autophagy is considered to be one of the major pathways in cancer that enables tumor growth and progression. The exact role played by autophagy in OSCC still needs to be comprehensively elucidated. More research on the role of this complex pathway in OSCC could provide an opportunity to develop novel and more efficient therapeutic strategies for OSCC patients.
